# The Impact of Co-Occurring Behavioral and Emotional Problems on the Quality of Life of Caregivers of Autistic Children: A Preliminary Study

**DOI:** 10.3390/jcm14103319

**Published:** 2025-05-09

**Authors:** Giulia Marafioti, Lilla Bonanno, Adriana Piccolo, Fabio Mauro Giambò, Viviana Lo Buono, Marcella Di Cara, Carmela De Domenico, Alessia Fulgenzi, Simona Leonardi, Caterina Impallomeni, Emanuela Tripodi, Angelo Quartarone, Francesca Cucinotta

**Affiliations:** Istituti di Ricovero e Cura a Carattere Scientifico (IRCCS) Centro Neurolesi “Bonino Pulejo”, S.S 113 Via Palermo C. da Casazza, 98124 Messina, Italy; giulia.marafioti@irccsme.it (G.M.); lilla.bonanno@irccsme.it (L.B.); fabio.giambo@irccsme.it (F.M.G.); viviana.lobuono@irccsme.it (V.L.B.); marcella.dicara@irccsme.it (M.D.C.); carmela.dedomenico@irccsme.it (C.D.D.); alessia.fulgenzi@irccsme.it (A.F.); simona.leonardi@irccsme.it (S.L.); caterina.impallomeni@irccsme.it (C.I.); emanuela.tripodi@irccsme.it (E.T.); angelo.quartarone@irccsme.it (A.Q.); francesca.cucinotta@irccsme.it (F.C.)

**Keywords:** autism spectrum disorder, levels of support, quality of life, caregiver

## Abstract

**Background:** Autism Spectrum Disorder (ASD) is a neurodevelopmental condition characterized by symptoms that vary in how severe they are. ASD individuals often present with psychiatric comorbidities that significantly impact their well-being and quality of life (QoL), with possible impacts on their family. **Aims:** This preliminary study aims to assess the impact of internalizing and externalizing behaviors, not closely related to the core symptoms of autism, on the QoL of their caregivers. **Method:** A pilot study was conducted with a sample of 58 children diagnosed with ASD and their caregivers, using the Quality of Life in Autism (QoLA) questionnaires for caregivers and the Child Behavior Checklist (CBCL) to evaluate the children’s behavioral and emotional problems. **Results and Conclusions:** The results revealed significant differences in the mean age of the children (3.32 ± 0.88 vs. 8.47 ± 2.51 years) and caregivers (35.68 ± 7.57 vs. 40.42 ± 6.43 years), with a notable impact of specific behavioral issues, such as attention, aggression, and externalizing behaviors, on the QoL of caregivers. In younger children, caregivers’ QoL was positively correlated with their age, with sleep problems being the primary source of stress. In older children, a negative correlation was found between caregivers’ age and their QoL, with conduct and social problems in children having a negative effect on caregivers’ well-being. These findings highlight the importance of targeted interventions to mitigate the impact of these factors on the QoL of caregivers of ASD children.

## 1. Introduction

Autism Spectrum Disorder (ASD) is a highly heterogeneous neurodevelopmental disorder, with severity defined based on the level of support required for daily functioning. The Diagnostic and Statistical Manual of Mental Disorders, Fifth Edition (DSM-5) [1], describes ASD as characterized by social and communication difficulties, rigid or repetitive behaviors, atypical interests, and problems related to sensory processing. In this edition, sensory characteristics were finally considered, and atypical responses to sensory stimuli, such as the presence of hyper- or hypo-reactivity to sensory input, or unusual interests in sensory aspects of the environment, were included in the diagnostic criteria. However, autistic individuals often experience co-occurring psychiatric disorders [2], which significantly impact their daily lives, well-being, and need for support. Psychiatric symptoms and behavioral disorders are frequently observed in ASD individuals, with approximately two-thirds reported to have at least one associated mental health condition [1]. Given the complexity and overlap between core ASD symptoms and psychiatric comorbidities, understanding their combined impact is crucial for developing comprehensive and individualized intervention strategies. Assessing the repercussions of potential comorbidities on the family system could help clinicians better evaluate the therapeutic or support interventions necessary to improve the quality of life (QoL) for the entire family unit [3,4]. QoL is a multidimensional concept where physical, psychological, and social components are influenced by personal characteristics and environmental variables [5,6].

Parents of ASD children may experience significant stress due to the severity and long-lasting nature of the condition, accompanying health issues, the necessity for intensive interventions for the child, and challenges in accessing required services [7]. The existing literature shows higher levels of stress, greater psychological distress and depressive signs, and higher rates of physical and mental health problems in caregivers of ASD children [8].

This can have a significant economic impact on the families involved, as noted by Cidav (2012) [9]. Parents of autistic children often report a lower quality of life compared to parents of typically developing children and those of children with other disabilities. [10,11]. The psychological and economic burdens can deeply influence family dynamics, leading to reduced social participation, isolation, and strained marital relationships, further exacerbating caregiver stress and impacting the overall family well-being [12].

ASD children may experience meltdowns and shutdowns in response to situations of stress or sensory and emotional overload [13]. A meltdown represents an intense and sudden emotional reaction, characterized by visible agitation, yelling, crying, or impulsive behaviors. This response is often triggered by strong sensory stimuli or sudden changes in routine. In contrast, a shutdown is a withdrawal response, during which the child appears distant, avoids eye contact, and reduces verbal communication. A shutdown is a strategy to cope with sensory or emotional overload, allowing the child to retreat and recharge to feel safe. These episodes may appear less noticeable than a meltdown, but they are no less significant in terms of their impact [13].

These reactions are natural for children with ASD and require understanding and support. Providing a calm and safe environment, reducing stressors, and using effective communication techniques can help to better manage these situations and promote the child’s well-being.

Raising a child with ASD can cause stressful conditions primarily related to the child’s adjustment to routines, interaction with health and education systems, coordination of multidisciplinary caregivers, and limited availability of resources [14].

This preliminary study aims to investigate whether the core symptoms of autism are the primary factors influencing the perceived QoL of parents of ASD, or if internalizing and externalizing behaviors also contribute significantly to this impact. Through this investigation, the goal is to gain a deeper understanding of the specific factors whether the core characteristics of autism or the secondary behaviors that contribute to the challenges faced by parents, and to inform the development of more targeted and effective support strategies.

## 2. Materials and Methods

This prospective, cross-sectional, observational pilot study was performed in accordance with the Declaration of Helsinki and received the approval of the Ethics Committee of the IRCCS Sicilia Centro Neurolesi “Bonino-Pulejo”. This clinical study adheres to CONSORT guidelines [15]. Written consent from parents or legal guardians was obtained. After a complete neuropsychiatric examination, children who met the inclusion criteria were selected and recruited. To assess caregiver-perceived quality of life and children’s emotional and behavioral problems, we administered the Autism Quality of Life (QoLA) questionnaire and the Child Behavior Checklist (CBCL 1½–5y, CBCL 6–18y) to children aged 1½–5 years and 6–18 years.

### 2.1. Inclusion/Exclusion Criteria

Parents of children were eligible for the study if their child met the following conditions: (a) aged between 1 and 16 years; (b) a diagnosis of ASD established according to DSM-5 criteria by trained psychiatrists; (c) diagnosis confirmed through the Autism Diagnostic Observation Schedule, Second Edition (ADOS-2); (d) provision of informed consent by the parent or legal guardian; (e) absence of auditory, visual, or physical symptoms; (f) absence of significant medical conditions, including epilepsy.

Children were excluded from the study if they met any of the following conditions: (a) age outside the 1–16 year range; (b) failure to meet the DSM-5 diagnostic criteria for ASD; (c) failure to obtain informed consent from the parent or guardian; (d) presence of significant medical conditions, including sensory deficits, epilepsy, genetic syndromes, or traumatic brain injury.

### 2.2. Participants

The participants were recruited consecutively from the child neuropsychiatry clinic at the IRCCS Centro Neurolesi “Bonino Pulejo” of Messina, Italy, where they received a clinical diagnosis of ASD. The sample consisted of 68 children, divided into two groups based on age. The first group included children aged between 1.5 and 5 years (3.20 ± 0.83), while the second group included children aged between 6 and 18 years (8.47 ± 2.51). The mean age of the caregivers in the first group was 35.68 years (±7.57), whereas the caregivers in the second group had a mean age of 40.42 years (±6.43). In the first group, the caregivers consisted of 21 mothers and 13 fathers, while in the second group, there were 25 mothers and 9 fathers.

### 2.3. Instruments

The assessment was carried out using the following tools: The Quality of Life in Autism questionnaire (QoLA) is a tool used to evaluate the quality of life of parents with autistic children [16]. It has two parts: Part A assesses the parent’s general quality of life, including emotional well-being, physical health, social relationships, and living environment. Part B measures the specific impact of the child’s autism-related issues on the parent’s quality of life, identifying the most relevant difficulties and how they affect the caregiver’s well-being. The QoLA was created to provide a comprehensive assessment of the daily challenges faced by parents and to help develop more targeted interventions to improve their overall well-being. The Child Behavior Checklist is a widely used tool for assessing behavioral and emotional problems in children. There are two versions of the CBCL based on age: the CBCL 1½–5 for preschool-aged children and the CBCL 6–18 for school-aged children and adolescents. The CBCL 1½–5 is designed to gather information on a wide range of behavioral and emotional issues in young children, typically reported by parents or caregivers. It includes questions about the child’s behavior over the past two months, covering domains such as emotional reactivity, anxiety/depression, somatic complaints, withdrawn behavior, sleep problems, and attention issues. The responses help to identify potential problems and guide further evaluations or interventions. The CBCL 6–18 is designed for older children and adolescents and gathers information from parents or guardians. This assessment covers similar domains to the preschool version but also includes additional items relevant to the developmental stages of older children, such as social problems, thinking problems, and transgressive behaviors. The checklist offers a comprehensive profile of the child’s behavioral and emotional functioning, which can be valuable for making diagnostic decisions and planning treatments.

### 2.4. Statistical Analysis

The normality of the data distribution was assessed using the Shapiro–Wilk test. Continuous variables were expressed as mean ± standard deviation for normally distributed data, while non-normally distributed numerical data were presented as median and interquartile range (first and third quartiles). For inter-group comparisons, the Mann–Whitney U test or an unpaired two-tailed Student’s *t*-test was used to determine differences in neuropsychological test scores between the 1½–5 and 6–18 age groups. For intra-group analysis, the relationship between QOLA (A, B, and total) and neuropsychological test scores was evaluated using Spearman’s rank correlation or Pearson’s correlation depending on data distribution. Finally, a multiple linear regression analysis was performed on QOLA total and subscores (QOLA-A and QOLA-B) (dependent variables) and the influence of QI and ASD level. We applied a backward elimination stepwise procedure for the choice of the best predictive variables according to the Akaike information criterion (AIC). Before conducting Pearson correlations and multiple linear regression analyses, assumptions of linearity and homoscedasticity were verified. The analyses were conducted using the open-source R4.2.2 software package provided by the R Foundation for Statistical Computing, Vienna, Austria. A confidence level of 95% was established with a 5% alpha error. Statistical significance was determined at a *p*-value of less than 0.05.

## 3. Results

### 3.1. Inter-Group Analysis

Inter-group analysis showed significant differences in children’s age (mean ± SD: 3.32 ± 0.88 vs. 8.47 ± 2.51 years, *p* < 0.001), caregiver’s age (35.68 ± 7.57 vs. 40.42 ± 6.43 years, *p* = 0.009) and in some CBCL subscores (Table 1), in particular, in attention (empirically/raw) (*p* < 0.001), aggressive (empirically/raw) (*p* = 0.004), other (empirically/raw) (*p* < 0.001), external (empirically/raw) (*p* = 0.003), attention (empirically weighted/weighted) (*p* = 0.04), anxiety (DSM-weighted/weighted-oriented) (*p* = 0.002), and QI (*p* = 0.03). Data are presented as means ± standard deviations for normally distributed variables and medians (first and third quartiles) for non-normally distributed variables.

### 3.2. Intra-Group Analysis

Intra-group analysis highlighted significant relationships in each group. In the 1.5–5 age group, the analysis revealed a significant positive Pearson correlation between QOLA A and the age of adults (r = 0.54; *p* = 0.002). QOLA B showed a significant positive Spearman correlation with sleep (empirical) (r = 0.39; *p* = 0.02) and sleep (empirical-weighted) (r = 0.36; *p* = 0.03). A trend was observed between QOLA B and anxiety (DSM) (Spearman, r = 0.33; *p* = 0.05), anxiety (DSM-weighted) (Spearman, r = 0.31; *p* = 0.08), and oppositional behavior (DSM-weighted) (Spearman, r = 0.34; *p* = 0.05).

In the 6–18 age group, a significant negative Pearson correlation was found between QOLA A and the age of adults (r = −0.38; *p* = 0.02), while a significant negative Spearman correlation was found with conduct (raw-oriented) (r = −0.35; *p* = 0.04). A trend was also observed between QOLA A and conduct (weighted-oriented) (Spearman, r = −0.33; *p* = 0.05). Finally, a trend was found between QOLA total and social behavior (raw) (Spearman, r = −0.32; *p* = 0.07). Finally, the multiple linear regression did not identify any significant predictors in each group.

## 4. Discussion

This study aims to assess whether it is solely the core symptoms of autism that have a significant impact on the perceived QoL of parents of ASD children, or if internalizing or externalizing behaviors also play a significant role. The main goal was to explore in depth the connection between the behavioral and emotional difficulties of ASD children and the impact these have on the psychological and physical well-being of their parents. This type of analysis is crucial, as a better understanding of these connections allows for the development of targeted interventions that can alleviate the emotional and practical burden on caregivers, improving both their QoL and the management of the child’s challenges. In this way, more effective tools could be provided to support not only ASD children but also their families in the long term. This study divided participants into two age groups, autistic children aged 1½ to 5 years and autistic children aged 6 to 18 years, to assess potential behaviors differences and their impact on the QoL of parents. In the younger children (1½–5 years), sleep problems were strongly correlated with a negative impact on the caregivers’ QoL. This suggests that sleep disturbances, particularly common in early childhood, also significantly affect the quality of caregiver sleep, which impacts their psychophysical well-being. In fact, the study conducted by Lovell et al. (2021) [17] demonstrated that caregivers commonly experience physical health problems closely related to sleep problems. Objective measures revealed that caregivers had more night-time awakenings (WASO), took longer to fall asleep, and experienced worse sleep quality despite spending more time in bed. Although physical health problems were not directly linked to recorded sleep data, they were still associated with self-reported sleep disturbances, indicating that poor sleep contributes to a decline in caregiver health [3,18]. In the group of older children (6–18 years), conduct problems, such as aggressive behaviors and inattention, were the most frequently observed. This finding is consistent with other studies that show a higher incidence of these behaviors compared to early childhood [19,20,21,22]. Analysis of our results showed that challenging behaviors, such as aggression and attention deficit, in older autistic children have a significant impact on parents’ QoL. These behaviors are a major source of stress for caregivers. Indeed, aggression and attention deficits, while not core symptoms of autism, are behavioral issues that significantly affect caregiver QoL and are similarly impactful in other neurodevelopmental conditions [23].

Additionally, a trend was found between total QOLA and social behavior (raw) (r = −0.32; *p* = 0.07). Social problems in older children and adolescents, such as difficulty following rules or interacting appropriately with peers, may further increase parental stress [24,25]. Parents may often find it difficult to help their children understand social rules that become increasingly complex as they grow older. These data are widely confirmed by several studies which demonstrate how parents consider social impairment as one of the most stressful aspects of raising autistic children [26,27]. Moreover, the well-being of the caregiver seems to be influenced by the parents’ age. In the group of younger children (1.5–5 years), a significant positive correlation was found between the caregiver’s age and the perceived QoL (r = 0.54; *p* = 0.002). This indicates that, for this group, an older caregiver is associated with a better perception of QoL. Conversely, a significant negative correlation emerged between the caregiver’s age and QoL (r = −0.38; *p* = 0.02). In this case, an increase in the caregiver’s age is associated with a poorer perception of QoL. This finding, in line with other work [28], could be explained by a greater burden that older caregivers have to face, as advanced age can bring additional challenges. This suggests that younger parents, likely due to greater energy and availability, may better manage the needs of an autistic child in the early stages of development. However, for older parents, the growing responsibilities and complexities associated with raising an autistic child can be particularly challenging, affecting their well-being.

Overall, the results of this study, consistent with previous research [29,30], confirm that externalizing behaviors are among the main sources of stress for parents of autistic children. A significant result that reinforces the importance of externalizing factors on parents’ quality of life emerges from the regression analysis, which considered the levels of support for ASD and the cognitive functioning of children as independent variables.

The results did not highlight significant predictors, indicating that neither the levels of support for ASD nor the cognitive levels represent determinants of the quality of life perceived by parents. This result, in line with previous studies [31,32,33], underlines how the child’s behavioral problems are more likely related to the psychological well-being of the caregiver than to the clinical characteristics related to ASD or to cognitive functioning. This can lead to increased stress, anxiety and, often, to an experience of physical and psychological exhaustion [34,35,36]. Our study highlights that the severity of behavioral problems in autistic children has a significant impact on caregivers’ QoL, particularly during the second childhood phase. In light of these findings, future research and intervention strategies should prioritize the implementation of multidimensional approaches that transcend clinical contexts, encompassing the educational [37] and social [38] environment of the child.

## 5. Limitations

The main limitations of the present study are the small sample size and the lack of a control sample. Our findings are just the beginning of understanding family dynamics in families with autistic children. Future research needs to expand the scope and include qualitative perspectives to capture the intricacies of these individuals’ daily lives. Additionally, it would have been important to also consider other parent demographic variables (e.g., level of social support, number of other children, ethnicity, and socioeconomic level), as they may constitute greater vulnerability or protective factors against parental distress. It will be essential to delve deeper into how behaviors that most significantly impact parents’ QoL, particularly the externalizing behaviors we have identified, can affect parenting styles and the quality of parent–child interactions. Future research should explore how parents respond to and interact with their autistic child’s problem behaviors. To this end, observational methods, such as guided play assessments, could be used to provide a more nuanced view of parent–child dynamics and how these interactions affect parental well-being. This comprehensive approach will not only help us understand daily family dynamics better but also allow us to tailor interventions to support caregivers. A detailed analysis is crucial to understanding the real effects of behavioral issues in autistic children on parents’ educational strategies, especially in response to management difficulties. The integration of these methodologies could provide a more detailed and accurate understanding of family interactions, providing a solid basis for the development of targeted support strategies to effectively improve the QoL of these families.

## Figures and Tables

**Table 1:** Demographics and clinical characteristics of groups.

	Groups	
	1½–5 Years	6–18 Years
Age_young	3.32 ± 0.88	8.47 ± 2.51
Age adult	35.68 ± 7.57	40.42 ± 6.43
Anxious	3 (1.25–5)	4.5 (3–7)
Withdrawn	4.5 (2.25–6.75)	2.5 (1.25–5)
Attention	4 (2–6)	8.41 ± 4.34
Aggressive	11.5 (6.25–16)	6.5 (3–10)
Emotional	2.5 (1–6)	-
Somatic	2 (1–4)	-
Sleep	3 (1–5.75)	-
Other	12 (8–19.25)	4.5 (3–9.75)
Somatic	-	2 (1–3)
Social	-	5.50 ± 3.35
Thinking	-	3 (2–6.75)
Criminal	-	3 (1–3)
Internal	12.5 (9–19.75)	10.5 (5–14)
External	15 (9–22.5)	10 (4–13.75)
Other	15 (9.25–23.5)	-
Total	40 (31.25–62.5)	44.5 (25–64.25)
Affective	3 (1.25–5)	4.21 ± 3.72
Anxiety	4 (2–5.75)	3.91 ± 2.60
ADHD	5.5 (4–8.75)	6.38 ± 3.53
Pervasive	6 (4–11.9)	-
Somatic	-	0 (0–1)
Conduct	-	2 (0.25–3)
Oppositional	3.5 (2–6)	2.5 (1–4)
Emotionally Weighted	53 (50–65)	-
Anxious-Weighted	52 (50.25–59)	58 (52.25–63)
Somatic-Weighted	53 (50–62)	57 (51–61)
Sleep-Weighted	53 (50–61.25)	-
Withdrawn-Weighted	65 (57–72.25)	60 (54–69.5)
Attention-Weighted	59.09 ± 8.67	63.71 ± 9.94
Aggressive-Weighted	52.5 (50–59)	56 (51–62)
Social-Weighted	-	61.06 ± 7.54
Thinking-Weighted	-	58 (54–69.25)
Criminal-Weighted	-	57 (51–59)
Internal-Weighted	58.15 ± 12.51	59.65 ± 10.37
External-Weighted	54.85 ± 10.49	56.62 ± 9.79
Total-Weighted	57.79 ± 13.07	61.03 ± 10.50
Affective-Weighted	56 (51.25–63)	63 (56–65.75)
Pervasive	63 (56–74)	-
Anxiety=Weighted	54 (50–59.25)	63.5 (54.25–69.5)
ADHD-Weighted	53 (51–63)	58 (53–67.5)
Somatic-Weighted	-	50 (50–56.75)
Conduct-Weighted	-	54 (50.25–56)
Oppositional-Weighted	51.5 (50–59)	53.5 (51–58)
QOLA A	100.03 + 15.45	96.62 ± 19.49
QOLA B	75.59 ± 15.07	72.09 ± 18.48
QOLA TOT	175.62 ± 21.28	168.71 ± 28.50
QI	65.91 ± 18.23	75.50 ± 16.80
ASD Level	2.0 (1–2)	2.0 (1–2)

Legend: QI = Intelligence Quotient; ASD = Autism Spectrum Disorder; ADHD = attention deficit hyperactivity disorder.

## Data Availability

Data and materials related to this study are available upon reasonable request from the corresponding author.

## Abbreviations

The following abbreviations are used in this manuscript:

ASD	Autism Spectrum Disorder
QoL	Quality of life
QoLA	Quality of Life in Autism
CBCL	Child Behavior Checklist
DSM-5	Diagnostic and Statistical Manual of Mental Disorders, Fifth Edition
ADOS-2	Autism Diagnostic Observation Schedule, Second Edition
WASO	Wake After Sleep Onset
QI	Intelligence Quotient
ADHD	Attention deficit hyperactivity disorder

## References

[1] American Psychiatric Association (2013). Diagnostic and Statistical Manual of Mental Disorders.

[2] Waizbard-Bartov E., Fein D., Lord C., Amaral D.G. (2023). Autism Severity and Its Relationship to Disability. Autism Res..

[3] Vasilopoulou E., Nisbet J. (2016). The Quality of Life of Parents of Children with Autism Spectrum Disorder: A Systematic Review. Res. Autism Spectr. Disord..

[4] Maggio R., Turriziani L., Campestre C., Di Cara M., Tripodi E., Impallomeni C., Quartarone A., Passantino C., Cucinotta F. (2023). An Individual-Supported Program to Enhance Placement in a Sheltered Work Environment of Autistic Individuals Mostly with Intellectual Disability: A Prospective Observational Case Series in an Italian Community Service. Front. Psychiatry.

[5] Cummins R.A. (2005). Moving from the Quality of Life Concept to a Theory. J. Intellect. Disabil. Res..

[6] Cucinotta F., Lintas C., Tomaiuolo P., Baccarin M., Picinelli C., Castronovo P., Sacco R., Piras I.S., Turriziani L., Ricciardello A. (2023). Diagnostic Yield and Clinical Impact of Chromosomal Microarray Analysis in Autism Spectrum Disorder. Mol. Genet. Genom. Med..

[7] Vohra R., Madhavan S., Sambamoorthi U., St Peter C. (2014). Access to Services, Quality of Care, and Family Impact for Children with Autism, other Developmental Disabilities, and other Mental Health Conditions. Autism.

[8] Totsika V., Hastings R.P., Emerson E., Lancaster G.A., Berridge D.M. (2011). A Population-Based Investigation of Behavioural and Emotional Problems and Maternal Mental Health: Associations with Autism Spectrum Disorder and Intellectual Disability. J. Child Psychol. Psychiatry.

[9] Cidav Z., Marcus S.C., Mandell D.S. (2012). Implications of Childhood Autism for Parental Employment and Earnings. Pediatrics.

[10] Dey M., Paz Castro R., Haug S., Schaub M.P. (2019). Quality of Life of Parents of Mentally-Ill Children: A Systematic Review and Meta-Analysis. Epidemiol. Psychiatr. Sci..

[11] Weiss M.J. (2002). Hardiness and Social Support as Predictors of Stress in Mothers of Typical Children, Children with Autism, and Children with Mental Retardation. Autism.

[12] Anchesi S.D., Corallo F., Di Cara M., Quartarone A., Catalioto R., Cucinotta F., Cardile D. (2023). Autism and ADHD: A Literature Review regarding their Impacts on Parental Divorce. Children.

[13] Phung J., Penner M., Pirlot C., Welch C. (2021). What I Wish You Knew: Insights on Burnout, Inertia, Meltdown, and Shutdown from Autistic Youth. Front. Psychol..

[14] Twoy R., Connolly P.M., Novak J.M. (2007). Coping Strategies Used by Parents of Children with Autism. J. Am. Assoc. Nurse Pract..

[15] Eldridge S.M., Chan C.L., Campbell M.J., Bond C.M., Hopewell S., Thabane L., Lancaster G.A., PAFS consensus group (2016). CONSORT 2010 Statement: Extension to Randomised Pilot and Feasibility Trials. BMJ.

[16] Eapen V., Crnčec R., Walter A., Tay K.P. (2014). Conceptualisation and Development of a Quality of Life Measure for Parents of Children with Autism Spectrum Disorder. Autism Res. Treat..

[17] Lovell B., Elder G.J., Wetherell M.A. (2021). Sleep Disturbances and Physical Health Problems in Caregivers of Children with ASD. Res. Dev. Disabil..

[18] Tsai C.H., Chen K.L., Li H.J., Chen K.H., Hsu C.W., Lu C.H., Hsieh K.Y., Huang C.Y. (2020). The Symptoms of Autism Including Social Communication Deficits and Repetitive and Restricted Behaviors Are Associated with Different Emotional and Behavioral Problems. Sci. Rep..

[19] Caamaño M., Boada L., Merchán-Naranjo J., Moreno C., Llorente C., Moreno D., Arango C., Parellada M. (2013). Psychopathology in Children and Adolescents with ASD Without Mental Retardation. J. Autism Dev. Disord..

[20] Choi H., Kim J.H., Kim H., Cheon K.A. (2023). Identifying Major Predictors for Parenting Stress in a Caregiver of Autism Spectrum Disorder Using Machine Learning Models. Front. Neurosci..

[21] Sikora D.M., Hall T.A., Hartley S.L., Gerrard-Morris A.E., Cagle S. (2008). Does Parent Report of Behavior Differ Across ADOS-G Classifications: Analysis of Scores from the CBCL and GARS. J. Autism Dev. Disord..

[22] Farmer C.A., Aman M.G. (2011). Aggressive Behavior in a Sample of Children with Autism Spectrum Disorders. Res. Autism Spectr. Disord..

[23] Baker B.L., Blacher J., Olsson M.B. (2005). Preschool Children with and without Developmental Delay: Behaviour Problems, Parents’ Optimism and Well-Being. J. Intellect. Disabil. Res..

[24] Lecavalier L., Leone S., Wiltz J. (2006). The Impact of Behaviour Problems on Caregiver Stress in Young People with Autism Spectrum Disorders. J. Intellect. Disabil. Res..

[25] Osborne L.A., Reed P. (2009). The Relationship between Parenting Stress and Behavior Problems of Children with Autistic Spectrum Disorders. Except. Child..

[26] Bebko J.M., Konstantareas M.M., Springer J. (1987). Parent and Professional Evaluations of Family Stress Associated with Characteristics of Autism. J. Autism Dev. Disord..

[27] Kasari C., Sigman M. (1997). Linking Parental Perceptions to Interactions in Young Children with Autism. J. Autism Dev. Disord..

[28] Marsack-Topolewski C.N., Church H.L. (2019). Impact of Caregiver Burden on Quality of Life for Parents of Adult Children with Autism Spectrum Disorder. Am. J. Intellect. Dev. Disabil..

[29] Bauminger N., Solomon M., Rogers S.J. (2010). Externalizing and Internalizing Behaviors in ASD. Autism Res..

[30] Hastings R.P., Taunt H.M. (2002). Positive Perceptions in Families of Children With Developmental Disabilities. Am. J. Ment. Retard..

[31] Bishop S.L., Richler J., Cain A.C., Lord C. (2007). Predictors of Perceived Negative Impact in Mothers of Children with Autism Spectrum Disorder. Am. J. Ment. Retard..

[32] Hastings R.P., Kovshoff H., Ward N.J., degli Espinosa F., Brown T., Remington B. (2005). Systems Analysis of Stress and Positive Perceptions in Mothers and Fathers of Pre-School Children with Autism. J. Autism Dev. Disord..

[33] Manning M.M., Wainwright L., Bennett J. (2011). The Double ABCX Model of Adaptation in Racially Diverse Families with a School-Age Child with Autism. J. Autism Dev. Disord..

[34] Brobst J.B., Clopton J.R., Hendrick S.S. (2009). Parenting Children with Autism Spectrum Disorders. Focus Autism Other Dev. Disabil..

[35] Hoffman C.D., Sweeney D.P., Hodge D., Lopez-Wagner M.C., Looney L. (2009). Parenting Stress and Closeness. Focus Autism Other Dev. Disabil..

[36] Schieve L.A., Blumberg S.J., Rice C., Visser S.N., Boyle C. (2007). The Relationship Between Autism and Parenting Stress. Pediatrics.

[37] Folostina R., Dumitru C., Iacob C.I., Syriopoulou-Delli C.K. (2022). Mapping Knowledge and Training Needs in Teachers Working with Students with Autism Spectrum Disorder: A Comparative Cross-Sectional Investigation. Sustainability.

[38] Little L.M., Cohen S.R., Tomchek S.D., Baker A., Wallisch A., Dean E. (2023). Interventions to Support Social Participation for Autistic Children and Adolescents in Schools (2013–2021). Am. J. Occup. Ther. Off. Publ. Am. Occup. Ther.Assoc..

